# Physicians' communication with patients about adherence to HIV medication in San Francisco and Copenhagen: a qualitative study using Grounded Theory

**DOI:** 10.1186/1472-6963-6-154

**Published:** 2006-12-04

**Authors:** Toke S Barfod, Frederick M Hecht, Cecilie Rubow, Jan Gerstoft

**Affiliations:** 1 Department of Infectious Diseases, Rigshospitalet, University of Copenhagen, Copenhagen, Denmark; 2 Positive Health Program, San Francisco General Hospital, San Francisco, California, U.S.A., University of California, San Francisco, USA; 3 Department of Anthropology, University of Copenhagen, Denmark

## Abstract

**Background:**

Poor adherence is the main barrier to the effectiveness of HIV medication. The objective of this study was to explore and conceptualize patterns and difficulties in physicians' work with patients' adherence to HIV medication. No previous studies on this subject have directly observed physicians' behavior.

**Methods:**

This is a qualitative, cross-sectional study. We used a Grounded Theory approach to let the main issues in physicians' work with patients' adherence emerge without preconceiving the focus of the study. We included physicians from HIV clinics in San Francisco, U.S.A. as well as from Copenhagen, Denmark. Physicians were observed during their clinical work and subsequently interviewed with a semi-structured interview guide. Notes on observations and transcribed interviews were analyzed with NVivo software.

**Results:**

We enrolled 16 physicians from San Francisco and 18 from Copenhagen. When we discovered that physicians and patients seldom discussed adherence issues in depth, we made adherence communication and its barriers the focus of the study. The main patterns in physicians' communication with patients about adherence were similar in both settings. An important barrier to in-depth adherence communication was that some physicians felt it was awkward to explore the possibility of non-adherence if there were no objective signs of treatment failure, because patients could feel "accused." To overcome this awkwardness, some physicians consciously tried to "de-shame" patients regarding non-adherence. However, a recurring theme was that physicians often suspected non-adherence even when patients did not admit to have missed any doses, and physicians had difficulties handling this low believability of patient statements. We here develop a simple four-step, three-factor model of physicians' adherence communication. The four steps are: deciding whether to ask about adherence or not, pre-questioning preparations, phrasing the question, and responding to the patient's answer. The three factors/determinants are: physicians' perceptions of adherence, awkwardness, and believability.

**Conclusion:**

Communication difficulties were a main barrier in physicians' work with patients' adherence to HIV medication. The proposed model of physicians' communication with patients about adherence – and the identification of awkwardness and believability as key issues – may aid thinking on the subject for use in clinical practice and future research.

## Background

The prognosis of HIV-infected people has improved dramatically since the introduction of Highly Active Antiretroviral Therapy (HAART) [1,2]. However, a large proportion of patients have poor adherence to HAART [3,4], and this is the main reason for treatment failures and the development of virological resistance [2,4,5]. Several factors are related to non-adherence, especially patient-related factors such as depression, abuse, and weak social support, but also regimen complexity, patient's lack of trust in the treatment, and poor patient-physician relations [3,6,7]. When looking at physician factors, we find that experienced physicians achieve better patient adherence [8], and that trusting patient-physician relations [9,10] and open communication [11] are associated with better adherence to HAART. In interviews, patients also stress that communication with physicians is important in maintaining adherence to HAART [12] as well as other diseases [5,13]. Accordingly, guidelines for treating patients with HAART recommend that adherence be addressed at all follow-up visits to prevent treatment failure [2,14]. The majority of physicians dealing with HIV also report that they do so [15-18].

Physicians' communication with patients about adherence to HAART can, however, be problematic. In descriptive questionnaire and interview studies physicians have identified lack of time and resources, as well as their own lack of training as the main barriers to their communication with HIV-positive patients about adherence [16-18]. Furthermore, a recent systematic review has concluded that two-way discussions and partnership in treatment decisions regarding medicine-taking in general most likely seldom take place [13]. To our knowledge, no observational study exploring physicians' communication with patients about adherence to HAART has been done and no analytical model of adherence communication has been developed.

The overall aim of this study was to observe and explore physicians' work with patients' adherence. During the study, communication emerged as a main issue. The aim of the present analysis therefore is to describe, conceptualize, and interpret the communication patterns of physicians when they discuss with patients about treatment adherence, and to explore the difficulties they face and the ways they handle them. During this process we developed a proposed model of four basic steps and three main factors/determinants of physicians' adherence communication. Since most HAART adherence studies have been done in the U.S. [19], we wanted to explore the possible role of contextual factors and included a U.S. setting as well as a setting outside the U.S.

## Methods

### Qualitative research approach

We used interviews to explore how physicians understand and make sense of their own situation and behavior [20], and we used direct observation to transcend their own understanding [21,22] in a multidisciplinary approach based on medical qualitative research methods [23]. Inspired by comparative anthropology [21,24], we studied both US and European physicians to explore the role of contextual factors.

We used a Grounded Theory methodology to interpret our findings. Grounded Theory is a systematic method for generating concepts from data and formulating relevant hypotheses about the determinants and consequences of the "Basic Social Processes" by which people handle their main concerns [22,25]. The ultimate goal is to generate theory or build a model that "works, fits, and is relevant," [22]. The model is modifiable and not a validated fact [22,25]. In Grounded Theory, data are analyzed concurrently with data collection and the main issues are allowed to emerge during coding and conceptualization of data [22,25]. In Grounded Theory, the coding process is divided into two or three stages. First, coding is "open" and in the end it becomes "selective" [22,25]. Strauss and Corbin furthermore describe an intermediate "axial" coding stage, where the concepts are organized into a "conditional matrix" [25]. In this study, we used much of Strauss and Corbin's practical advice for the analytic process, while following Glaser's advice not to force the data into a predefined "conditional matrix."

Prior to the interviews, we were familiar with the classical health behavior models [26], some main adherence theories [27,28], the basics of physician-patient communication [29,30], and with guidelines for HAART adherence counseling [2,31]. However, in Grounded Theory pre-formulated concepts and theories are only used to "sensitize" the researcher, as all concepts must earn their relevance through constant comparison with data [22,25].

### Settings and physicians

We chose San Francisco and Copenhagen, as both cities may be expected to provide "state of the art" services. We included five large outpatient clinics, three in San Francisco: University of California, San Francisco (UCSF), San Francisco General Hospital (SFGH), Mission Neighborhood Health Center (MNHC); and both of the two existing clinics in Copenhagen: Rigshospitalet (RH) and Hvidovre Hospital (HH).

In San Francisco 16 out of 23 eligible physicians participated, in Copenhagen 18 out of 19. Of the seven non-participants in San Francisco, three never responded to our e-mail, three consented but were not included due to illness or time constraints, and one declined without explanation. The non-participating physician in Copenhagen was excluded because of time constraints. Table 1 shows the characteristics of the participating physicians. An additional file includes calculated percentages of physicians with each characteristic (Additional file 1).

### Procedures and analysis

Data were collected from December 2001 to August 2003. We invited all physicians employed at the clinics by e-mail or posted letter and participants signed a consent form. We gave patients an information sheet and all participating patients gave explicit verbal consent. The first author observed each physician's consultations during one workday and simultaneously took notes on the physician's verbal and some non-verbal communication. To minimize intrusion and ensure confidentiality, and to facilitate participation of all physicians, we did not tape-record or film the consultations. Subsequently, a qualitative, semi-structured interview [20] with the physician was done about how he or she had perceived and worked with patients' adherence that day (interview guide enclosed as Additional file 2). Physicians were asked how they had assessed and enhanced each patient's adherence, how they would explain each patient's degree of adherence, and how they recalled and interpreted their own communication with the patient about adherence. For validation, physicians were invited to comment on the researcher's noted observations and immediate interpretations, as recommended by Kvale [20]. These comments were included as further data to verify, correct, and broaden the observations and interpretations of the researcher. Interviews with physicians lasted about one hour, were audio-recorded and transcribed verbatim. The handwritten notes on observations were typed into a word-processing program within one day of the interview.

At the end of interviews, physicians were asked if they felt they had changed behavior due to the presence of the observer. Generally, physicians stated that they were used to having students observe their work and that the observer's behavior was easy-going and non-intrusive. Fourteen of the physicians stated that there was no influence from the observer, 13 stated that they had been conscious of the presence of the observer, especially at the beginning of the day, although it did not change their adherence communication, and 6 said they had probably focused a little more on adherence than they usually would. One physician's answer to this question was not recorded.

In the analysis, all interviews were replayed on audio and all data were re-read several times. The transcribed interviews, as well as the notes on observations, were used as data. First, a brief summary of the observations and interview with each physician was written within one day of the interview. Then, during open coding, all notes on observations and the transcribed interviews were fragmented into meaning units (a few sentences or a paragraph), which were labeled with one or more concepts or statements. Concepts where developed both from the interviewed physicians' own statements and the researcher's interpretations. During the entire coding process, analytical memos on concepts were written, concepts were renamed, units of text were recoded, and recurring themes were noted [22,25]. After the open coding, we narrowed our focus to the communication process and related concepts to each other during selective coding. Theoretical relations between concepts (e.g., that XX leads to YY) were developed from analysis of observations as well as from interviews. During this process, several alternative models were developed and explored, and finally we ended up with a simple four-stage three-factor model. We did not arrive at a single core concept. For practical handling of the large amounts of conceptualized text, NVivo software was used (Version 2.0, by QSR International Pty).

We did the sampling at five different clinics in two different cities to allow for the role of contextual factors to emerge. However, as the similarities of communication patterns at the different sites were much larger than the differences, contextual factors came to play only a minor role in the final analysis.

The first author, a bilingual physician with training in qualitative research, did all the observations and interviews as well as the primary data analysis. Data collection and analysis were continuously checked with the co-authors (two physicians and one anthropologist) and with external physicians and methodologists to validate findings and broaden the analysis by incorporating viewpoints from multiple disciplines [23]. The Institutional Review Board at UCSF approved the study.

### Patients and consultations observed

In total, 183 consultations were observed. In San Francisco, 49 consultations with patients receiving HAART were observed as well as 11 consultations with patients not currently on treatment. In Copenhagen, the corresponding numbers were 95 and 28.

In San Francisco, we observed consultations with 42 men, 6 women and 1 transgender currently on HAART. In Copenhagen, the corresponding numbers were 78, 17 and 0. In San Francisco, 27 were of Caucasian origin, 22 were not. In Copenhagen, there were 77 and 18, respectively. We aimed to minimize patient dropout and to allow an undisturbed interaction between patient and physician. Therefore we only collected the directly observable data on patients, and did not ask about patients' age, mode of transmission, housing situation or drug use habits.

However, we roughly estimate that in San Francisco 60% of the included patients had been infected through homosexual practices, 15% heterosexually, and 20% through intravenous drug abuse, whereas the corresponding estimates in Copenhagen are 50%, 40% and 5%, respectively [3]. We further estimate that roughly 20% of included patients in San Francisco were regular users of illegal drugs other than marijuana and that 10% were homeless, whereas the corresponding numbers in Copenhagen are 10% and <1%, respectively.

Approximately 10% of consultations were not observed, as requested by patient or physician. Patients and physicians most often explained that this was because sexual issues were to be discussed. Only very few patients gave other explanations or no explanation.

## Results

### Overview of findings and comparison of San Francisco and Copenhagen

The main communication patterns were similar in San Francisco and Copenhagen. In both settings, in-depth discussions between patient and physician were rare, although adherence was mentioned in more than half of the consultations. Patients hardly ever brought up the subject themselves and when physicians brought up the subject, patients usually gave brief answers that often had low believability. It emerged that physicians had individual communication patterns, which were not only determined by their perceptions about patients' adherence, but also strongly influenced by their perceptions about the awkwardness of discussing adherence with patients and their perceptions about the believability of patients' statements on adherence. These three aspects of physician perceptions depended on the general attitudes of the physicians as well as the specific circumstances with a specific patient (e.g., when a physician suspected that a patient was non-adherent, it was a function of his or her general suspicion of non-adherence as well as the observation of specific clues in this specific patient). The physicians' age, gender, experience, and education did not emerge as main determinants of their communication with patients about adherence to HAART.

**Figure 1:**
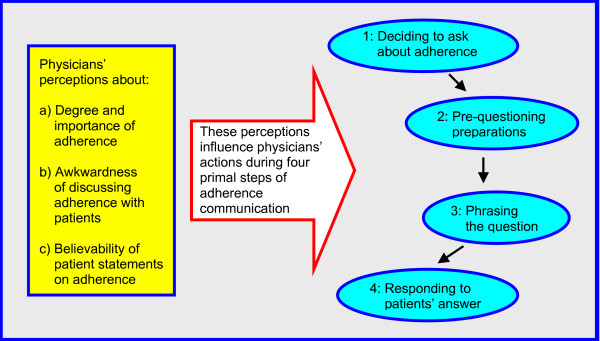
**Model of physicians' adherence communication (simple version)**. Physicians go through four steps when communicating with patients about adherence. The way physicians act in these is markedly influenced by three aspects of their perceptions (see figure 2 for an expanded version of the model).

**Figure 2:**
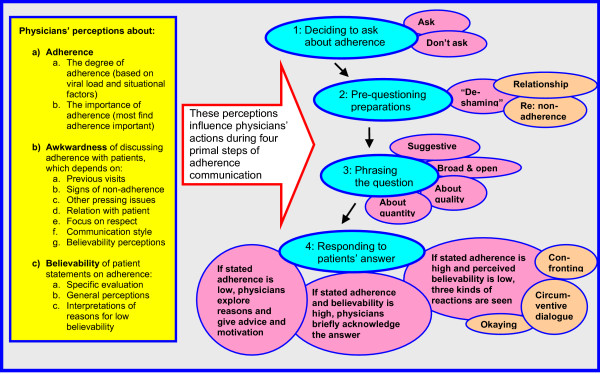
**Model of physicians' adherence communication (expanded version)**. Physicians go through four steps when communicating with patients about adherence. The way physicians act in these is markedly influenced by three aspects of their perceptions. Each of the four steps has its subcategories, and each of the three perceptions has its main determinants (see figure 1 for a simple version of the model).

We here propose a model of how physicians' perceptions of these three factors (adherence, awkwardness, and believability) shaped their decision to ask about adherence, their possible pre-questioning preparations, their phrasing of the question, and their response to the patient's answer. We present a simple outline of the model (Figure 1) and an expanded version with subcategories (Figure 2). The model is further explored in the body of this paper. We first describe the three perception factors and their main determinants (or "subcategories"). Then we describe the main ways that physicians act during the four steps in the communication process and how the three perception factors influence these actions. In turn, we briefly look at the consequences of these actions for the awkwardness, believability, and adherence information content of patient responses as perceived by physician and researcher.

We observed a few differences between San Francisco and Copenhagen in terms of adherence communication. Average consultations were longer in San Francisco than in Copenhagen (26 vs. 16 minutes) (Table 1) and the subject of adherence was mentioned in 36 of 49 (73%) consultations in San Francisco compared to 58 of 95 (61%) in Copenhagen. Adherence discussions were slightly more comprehensive in San Francisco, where a question style implying that the patient had missed some doses of medication was mainly observed, whereas a question style implying good adherence was mainly observed in Copenhagen (described in more detail later). The atmosphere seemed less formal in San Francisco than in Copenhagen, e.g., some physicians gave patients a hug or told them about incidents from the physicians' own private lives. Since the similarities between communication patterns in San Francisco and Copenhagen were so much larger than the differences, in this paper we will not further dwell on the differences.

### Factor A: Adherence perceptions

Physicians' communication with patients about adherence was – not surprisingly – strongly influenced by their perceptions about the patients' degree of adherence and the perceived importance of adherence.

Physicians determined the degree of adherence both from the treatment effect (viral load) as well as from situational factors. If the patient had a rising viral load, physicians would virtually uniformly be suspicious that the patient might have low adherence, especially if the viral load was rising from very low (i.e., "undetectable") levels. However, the interpretation of an undetectable (or otherwise stable) viral load varied considerably, since some would consider this proof that the patient was sufficiently adherent, whereas others would still be very alert for poor adherence. Physicians' interpretations of the patients' situational factors varied considerably. However, all physicians generally made an overall assessment based on the patient's lifestyle, abuse patterns, perceived personality, and timing of medication refills, and they listened to patients' statements regarding adherence.

Most physicians had the general perception that adherence was very important: "It's the most important limiting factor in treatment," (SF3) or "I do a lot, I think, around adherence issues 'cause the stakes are so high" (SF11). A few physicians, however, felt that there was no need to worry much about adherence, as long as the viral load was undetectable, and others did not worry if the patient already had multi-drug resistance and a high viral load. For example, one part of an interview went like this: "INT: Can you say more about to what degree [patients] are sufficiently adherent when they are undetectable? ... DR: Well, I mean what is the goal of anti-viral therapy? I guess it's to drive the virus to undetectable [...] INT: So you don't think they could be missing enough to be at risk of developing resistance? DR: I don't care. That's not a big worry to me – I'm not a big resistance-phobic person" (SF13). Physicians who did not consider adherence to be an important issue tended to communicate less with patients on the subject.

### Factor B: Perceived awkwardness of exploring adherence

Physicians seldom spontaneously declared that exploring adherence was an awkward thing to do. But when physicians were asked in the interview why they had touched on the subject the way they did, perhaps only superficially or not at all, they often explained that further explorations were unnecessary and also would have been too awkward: "Some patients can get a bit offended if you ask [about adherence]... They may feel that the trusting relationship is challenged... I remember one patient who got very defensive and said *'But you know that I have always taken the medicine, why do you now suddenly start sitting there saying things like that'*." (Cph8).

Physicians mainly perceived explorations into adherence to be too awkward if the patient had stated good adherence on previous visits: "It's the awkwardness of the repetition of the series of questions" (SF7). Physicians also perceived explorations to be awkward when there were no objective signs of non-adherence, when there were other pressing issues in the consultation, or if the physician perceived the relation with the particular patient to be difficult and fragile.

Explorations were also often considered awkward if the physician generally focused very much on showing patients respect and on avoiding creating feelings of guilt: "I think [the physician] being *in loco parentis *too much is not what adult [patients] are going to really be thrilled about. You're more apt to get positive results if you're trusting and a little lenient" (SF13), or "I'll rather praise people than make them feel guilty by insisting on exploring something that may not be working ideally, but which works okay" (Cph15).

Exploring adherence was not perceived as awkward if the physician had a "de-shaming" communication style (see below), did not worry about the patients' possible feelings of shame and the believability of the answer, or did not perceive the patient relation to need special nurturing.

### Factor C: Believability perceptions

Believability issues were also important during all four steps of physicians' communication strategies and were determined by the specific situation as well as the physician's general perceptions.

In the specific situation, the believability of a patient's claims of good adherence was evaluated by physicians from their independent assessment of the patient's degree of adherence (based on viral load and situational factors as described above), coupled with the patient's perceived general trustworthiness and the phrasing and tone of the patient's adherence statements. If the patient was very firm in his intonation or detailed in his description of medication intake, the patient's answer would more often be believed. If patients disclosed non-adherence, physicians practically always believed this, although they sometimes felt the non-adherence was understated.

Physicians differed in their general perceptions regarding believability. Some physicians felt that patient statements on adherence were generally believable: "I actually believe what patients tell me" (Cph1), and physicians could even seem torn between their suspicion of poor adherence and an almost moral obligation to trust patients. Others accepted low believability with ease: "It's ... in my opinion, one of the hardest things to get a truthful answer for (SF9).

The underlying reasons for low believability were explained by physicians in various ways. Quite often, low believability was explained by the patient's politeness or sympathy with the doctor: "Clients are very aware of what their doctors want to hear, particularly if they like their doctor" (SF9), or by the patient's shame: " [Admitting having missed doses] is an admission of failure. And then they think the doctor finds them stupid or not serious about it" (Cph13). Low believability of patients' answers was seldom attributed to poor memory or mental repression, though sometimes to "craziness" or unacceptable manipulation and arrogance: "I just don't want to sit there and be ridiculed ... that they just sit and decide they know better than me" (Cph14).

In the following, we will explore how physicians' perceptions of adherence, awkwardness, and believability influence the way physicians handle the four steps in the communication process.

### Step 1: Deciding whether to ask about adherence or not

Some physicians rarely asked about adherence, others asked only superficially, and very few asked most of their patients in depth. Physicians' decision to ask or not was largely determined by their perceptions of adherence, awkwardness, and believability. Patients hardly ever brought up the subject themselves.

Generally, physicians usually asked about adherence if they perceived a patient's adherence to be low and they perceived adherence to be an important issue.

However, if physicians perceived the specific patient's adherence to be good, or if they generally did not consider it a very important issue, they often felt that it was not necessary to ask, and also that it would have been awkward to do so: "The reason that I do not ask more [... about adherence] could be that it feels unnecessary. And it could perhaps seem like a silly question, sometimes" (Cph1).

Physicians sometimes would not ask about adherence if they had very low trust in the believability of patients' answers on this issue: "To ask *'Do you sometimes forget to take your medication' *can be used for nothing ... There are these studies we have seen, showing it is useless. It's fifty-fifty whether they answer yes or no – no matter what situation they have been in" (Cph7).

On the other hand, physicians could also be led to abstain from asking about adherence if they trusted the patients so much that they even expected them to spontaneously tell about possible adherence problems: "I will not ask ... everybody whether they have ... forgotten a dose on a single occasion ... this of course has to do with that I generally believe ... patients' bring up their problems to surface" (Cph1).

### Step 2: Pre-questioning preparations

Physicians were asked if they did anything to facilitate communication about adherence. Most answered that they – even before asking about adherence – tried to create a trusting, informal, and friendly atmosphere. Physicians often felt this "de-shamed" patients and made it easier for patients to be honest, e.g., about non-adherence. Many physicians were also observed to have an informal body language, to use slang and jokes, and to chat with patients about private things, like how the patient had spent his vacation.

Physicians usually popped adherence questions abruptly without warning. Only when physicians were very aware of awkwardness and the need to promote believability did they prepare patients for the question with a "warning shot," e.g. by referring to prior discussions or the results of recent blood tests. One physician was also observed to "de-shame" a patient by generalizing adherence problems prior to asking about adherence, saying: "Most people find it hard to remember taking the medication" (Cph8). This remark did trigger disclosure of non-adherence and other physicians referred to prior successful use of similar phrases.

### Step 3: Phrasing the question

When physicians individualized questions and picked from a broad palette of question styles and content it seemed to facilitate elaborate answers. However, most physicians used a favorite phrase with most patients.

#### Question styles

*Broad and open questions* were common. Physicians asked, "How are you doing with the medication?" (SF11) or "How is it going with taking the medication?" (SF15). Patients' first answers were often only superficial or not about adherence. Only when physicians gave very much priority to adherence, would they follow-up with questions that were more specific.

*Suggestive questions* were also common. Suggestive questions implying that some doses might have been missed could be, "How many doses have you missed in the last 14 days" (SF14). Such questions were mainly asked when physicians were very focused on the need to promote believability. Physicians felt such phrasing made it less awkward for people to admit having missed doses, because "this means everybody is missing" (SF14). On the other hand, suggestive questions implying good adherence could be, "You don't have any problems taking your medication, do you?" (Cph11). This kind of phrasing was mainly used when physicians were less focused on the need to promote believability and more focused on maintaining a respectful, non-awkward communication in general. Such phrases seemed to function mainly as a reminder to the patient of the importance of adherence and less as a facilitator of in-depth dialogue on the subject.

The tone of questions was mentioned by a few physicians who focused a lot on believability: "I always ask to what degree they're taking their pills and I try to do it in a low-key manner – kind of like offhand – so that my patients have an absolute sense that they can tell me everything" (SF1).

#### Content of questions

*Questions about the quantity of missed doses *were common when physicians perceived adherence to be important. These questions were used both to assess adherence and to remind the patient of its importance. Different degrees of specificity in number and time range were addressed, though a time range of two weeks was often used. Answers to these questions were often vague and their believability was often not convincing both to the physician and the observer.

*Questions about the qualitative adherence-related aspects of medication intake *were mainly asked when adherence was perceived to be an important but potentially awkward issue. These seemed less awkward to ask than questions about the quantity, and the answers seemed more believable. Three main topics were addressed:

* Knowledge of the regime: Whether patients could describe their regime was routinely checked by some: "I want to know what they are really taking, because... so many times they are not taking what is [written] on the bottle" (SF7).

* Motivation for treatment and adherence: This was mainly asked about by checking for side effects, which were often perceived to be the main motivational barrier to treatment. The patients' perception of positive treatment effects or their motivation for adherence was very seldom asked about.

* Behavioral patterns. Only when physicians gave adherence very high priority and they were very aware of the awkwardness of the subject did they ask about the routines patients had or could develop for taking and remembering the medication, and how they handled difficult adherence situations. Patients did, however, seem to talk more freely about these practical problems with adherence and seemed to become aware of new solutions.

### Step 4: Responding to patients' answers – handling varying degrees of believability

#### Responses to patients stating good adherence with high believability

When physicians perceived the believability of a statement on good adherence to be high, they would usually briefly acknowledge the answer, perhaps with praise, a warning about the possible consequences of non-adherence, or a question about side effects. Many physicians felt that a further exploration of the patient's adherence strategies would be awkward and unnecessary in this situation.

#### Responses to patients stating good adherence with low believability

Physicians responded to patients' statements of good adherence with low believability in three ways: *Okaying*, *circumventive dialoguing*, and *confronting*.

*Okaying the answer *despite its low believability was mainly done when physicians thought adherence was not that important, or that further explorations would be awkward, mainly because the relation to the patient was fragile. One physician more generally okayed patient statements even when they had low believability: "It was the message I wanted to send – that they can answer me whatever they want" (Cph15).

*Circumventive dialoguing *is here defined as continuing the communication on adherence without drawing attention to the possible low believability of patient statements. One important way to do circumventive dialoguing was to address the qualitative adherence-related aspects of medication intake instead of the quantity of missed doses, e.g., by asking what time of the day the medicine was taken, whether it was taken with food, etc. Another kind of circumventive dialogue was to re-ask closed questions about occurrence of missed doses, but with altered specificity regarding the time frame or number of missed doses. This was several times observed to elicit otherwise hidden non-adherence. For example, one dialogue went like this (SF2):

"Any problems with the medicine?"

"No."

"You take them all?"

"Yes, the 3TC, the Viramune... and the eeh, Epivir."

"Any problems taking them?"

"No."

"You took them this morning?"

"No man! I did not take them this morning!"

*Confronting low believability *covers a range of reactions from subtle signals of doubt to clear expressions of anger. For example, physicians confronted patients without being aggressive by stating that the patient's rising viral load without mutations was most easily explained by low adherence. Sometimes physicians explicitly asked for honesty. When physicians perceived low believability as unacceptable they were sometimes observed to shame the patient for lying or to get upset and angry, i.e., they displayed a raised voice and flushing skin.

#### Responses to patients stating poor adherence

Physicians virtually always believed in statements of poor adherence. Physicians explored the underlying reasons for poor adherence and attempted to assist with behavioral advice or they tried to strengthen motivation for adherence through information, condemnation, or shaming of the patient.

## Discussion

We found that physicians' communication with patients about adherence was often awkward and superficial, even when physicians tried to create a friendly atmosphere. To "de-shame" patients regarding poor adherence was an important, but underused strategy for facilitating communication on the subject. Physicians' interpretation of the believability of patients' statements on adherence was another major factor in the communication process. We developed a simple four-step model of physicians' communication with patients about adherence, where the content of each step depends on the physician's perception of three things: adherence, awkwardness, and believability. The main communication patterns were similar in San Francisco and Copenhagen, although a question style implying poor adherence was mainly observed in San Francisco and the adherence discussions in San Francisco were slightly more comprehensive than in Copenhagen.

To our knowledge, this is the first observational study to provide a model of physicians' communication with patients about adherence to HAART and the first study to propose a conceptualization of its main determinants.

The main weaknesses of this kind of study are that the analytical model cannot be interpreted as validated fact [20,22,23] and that the descriptive aspects cannot be generalized to other settings. San Francisco and Copenhagen are not typical HIV treatment sites, e.g., because of their high research priorities. However, the conceptual products of Grounded Theory methodology should have good a "fit" within context and can also sensitize physicians and researchers in other settings to the basic social processes discovered, although the specified processes may be less prevalent elsewhere [22]. Thus, the findings may be relevant in non-HIV settings as well.

It is a possible source of bias that the observation itself may have made physicians focus more than usual on adherence, despite their explicit statements to the contrary [32], and may have made the consultations more awkward. However, even though observations only lasted half a day to one day, there were very few indicators that the observed consultations were not "typical." Another limitation is the non-inclusion of patient's viewpoints, although our findings are supported by others who have interviewed patients [33-40].

Even in the expanded outline of the model with subcategories (Figure 2), we have not included the connections between specific perceptions and specific behaviors, as it would make the figure overly complex. This complexity of the full analysis may be viewed as a weakness of the study. However, but we believe the simple model (Figure 1) conveys the main messages.

The aforementioned minor differences in communication patterns between physicians in San Francisco and Copenhagen may be tentatively explained by some differences in context. As compared to Copenhagen, the clinics in San Francisco had longer consultations, much less follow-up by nurses, and a patient population with more homelessness and drug abuse. San Francisco, moreover, traditionally has a strong gay grass-roots HIV movement and a political HIV commitment among physicians, possibly linking physicians there very close to their patients. There may also be a general American tendency to openly sharing feelings [41]. Furthermore, some eligible physicians in San Francisco did not participate, leaving a selected sample to be studied. All this may contribute to the slightly more comprehensive adherence discussions and more consciously developed communication strategies observed in San Francisco than in Copenhagen.

Previous interview studies have highlighted lack of time, resources, education, and experience as the barriers to physicians' work with patients about adherence to HAART [15-18]. Our study highlights communication and the crucial role of adherence perceptions, awkwardness, and believability. These aspects of social interaction are often not given much attention in standard theories about health behavior [26], patient communication [29], and adherence support [2,14].

Recent studies find that HIV+ patients seldom tell physicians about adherence problems [34,35,42]. Our study points out physicians' difficulties of doing interviewing and counseling when patients are reluctant to tell about their problems. This is supported by a study of hypertension that points to the role of physicians' question styles in receiving believable information on adherence [43]. Our findings are also in line with an interesting study, which finds that when general practitioners meet a non-adherent diabetes patients, they tend to get frustrated and adopt a paternalistic attitude, and try to threaten and pressurize patients into becoming adherent [44].

The existing theory of motivational interviewing holds that assistance in behavior change should not primarily be done by giving advice and information, but rather by assisting patients in exploring their own priorities and in developing their own strategies for solving problems [30]. In line with this, recent guidelines for counseling about adherence to HAART [14] and other medications [5] stress that physicians should develop a partnership with patients and communicate in a non-judgmental way.

These recommendations for clinical practice are supported by our findings. However, we suggest they be supplemented with an enhanced focus on "de-shaming" techniques, the provision of a broadened palette of question styles, and some conscious strategies for sensibly handling low believability of patient statements on adherence. We believe this would make physicians better equipped for supporting patients' adherence.

Future research needs to challenge or verify our findings in other settings. Patients' perceptions of the awkwardness of discussing adherence and the background for low believability also need to be further explored.

## Conclusion

We found communication to be a main difficulty in physicians' work with patients' adherence to HAART. We developed a simple model of adherence communication, identifying three factors that influence how communication may proceed through four steps. This model – and the identification of awkwardness and believability as key issues in patient-physician communication on this subject – may aid analytical thinking on adherence communication for use in clinical practice and future research.

## Competing interests

The author(s) declare that they have no competing interests.

## Authors' contributions

TSB conceived the study, carried out all observations and interviews, and did the primary analysis and writing. CR, FMH, and JG participated substantially in planning, design, analysis, and writing. FMH and JG also facilitated contact to participants. All authors read and approved the final manuscript.

## Pre-publication history

The pre-publication history for this paper can be accessed here:



## Supplementary Material

Additional file 1 Table 1b: Characteristics of participating physicians, including calculated percentages of physicians with each characteristic. Click here for file

Additional file 2 Interview guide. Used for semi-structured interviews with physicians. Click here for file

## Figures and Tables

**Table 1:** Characteristics of participating physicians.

	**San Francisco (n = 16)**	**Copenhagen (n = 18)**	**Total (n = 34)**
**Gender**			
(female/male)	3/13	6/12	9/25
			
**Age distribution**			
30–39	6	3	9
40–49	5	9	14
50–59	4	4	8
60–69	1	2	3
			
**Specialty**			
Still in training	0	6	6
Infectious Diseases	4	12	16
Internal Medicine, incl. various specialties	9	0	9
Family Practice	3	0	3
			
**Clinic affiliation**			
San Francisco: SFGH/UCSF/MNHC^§^	10/3/3	-	10/3/3
Copenhagen: HH/RH^§^	-	7/11	7/11
			
**Minutes per routine consultation**			
(mean and range)	25.5 (20 – 30)	16 (12 – 20)	20.5 (12 – 30)
			
**Years of HIV ambulatory care**			
(mean and range)	10 (2 – 21)	9 (1 – 22)	9.5 (1 – 22)
			
**Ethnicity**			
(Caucasian/other)	13/3	18/0	31/3
			
**Working days per week in HIV ambulatory**			
(mean and range)	1.7 (1/2 – 4)	0.9 (1/2 – 1)	1.25 (1/2 – 4)
			
**Eligible physicians not participating**			
(total number)	7	1	8

^§^San Francisco General Hospital/University of California, San Francisco/Mission Neighborhood Health Center; Hvidovre Hospital/Rigshospitalet.
