# Supplementary material for: Physicians' communication with patients about adherence to HIV medication in San Francisco and Copenhagen: a qualitative study using Grounded Theory
Source: BMC Health Serv Res. 2006 Dec 4;6:154. doi: 10.1186/1472-6963-6-154 (PMC1702356; doi:10.1186/1472-6963-6-154)
Supplement: Additional file 1 — Table 1b: Characteristics of participating physicians, including calculated percentages of physicians with each characteristic. [file 1472-6963-6-154-S1.doc]

**Table 1b: Characteristics of participating physicians, including calculation of percentages of each characteristic**.

|  | **San Francisco (n=16)** | **Copenhagen (n=18)** | **Total**  **(n=34)** |
| --- | --- | --- | --- |
| **Female / male** (# and (%)) | 3 / 13 (19 / 81) | 6 / 12 (33 / 66) | 9 / 25 (26 / 74) |
| Age distribution (# and (%)) 30-39  40-49  50-59  60-69 | 6 (38)  5 (31)  4 (25)  1 (6) | 3 (17)  9 (50)  4 (22)  2 (11) | 9 (26)  14 (41)  8 (24)  3 (9) |
| **Specialty** (# and (%))  Still in training  Infectious Diseases  Internal Medicine, incl. various specialties  Family Practice | 0 (0)  4 (25)  9 (56)  3 (19) | 6 (33)  12 (66)  0 (0)  0 (0) | 6 (18)  16 (47)  9 (26)  3 (9) |
| **Clinic affiliation** (# and (%))  San Francisco: SFGH / UCSF / MNHC^§^  Copenhagen: HH / RH^§^ | 10 / 3 / 3  - | -  7 / 11 | (29 / 9 / 9)  (21 / 32) |
| **Minutes per routine consultation** (mean and range) | 25,5 (20 - 30) | 16 (12 - 20) | 20,5 (12 – 30) |
| Years of HIV ambulatory care (mean and range) | 10 (2 - 21) | 9 (1 - 22) | 9,5 (1 - 22) |
| **Caucasian / other** (# and (%)) | 13 / 3 (81 / 19) | 18 / 0 (100 / 0) | 31 / 3 (91 / 9) |
| **Working days per week in HIV ambulatory** (mean and range) | 1,7 (½ - 4) | 0,9 (½ - 1) | 1,25 (½ - 4) |
| **Eligible physicians not participating** (# and (% of all eligible)) | 7 (30) | 1 (5) | 8 (19) |

^§^San Francisco General Hospital / University of California SF / Mission Neighbourhood Health Centre; Hvidovre Hospital / Rigshospitalet.
