# Supplementary material for: Physicians' communication with patients about adherence to HIV medication in San Francisco and Copenhagen: a qualitative study using Grounded Theory
Source: BMC Health Serv Res. 2006 Dec 4;6:154. doi: 10.1186/1472-6963-6-154 (PMC1702356; doi:10.1186/1472-6963-6-154)
Supplement: Additional file 2 — Interview guide. Used for semi-structured interviews with physicians. [file 1472-6963-6-154-S2.doc]

# Guide to Interviews with Physicians

# Information to physicians before observations

“Thank you for your interest in our study.” Introduce self. Provide background and research interests. Provide overview of study. Ask if they would care to participate. Obtain consent.

(Can we wait ½ an hour after observation before interview?).

### Guide to interview

Intro: We are going to talk about the consultations and adherence and a bit about your background.

It is exploratory. Not with fixed questions.

You will remain anonymous. You may be anonymously quoted, such as “a female/male specialist in his/her 30’ies, with 2-5 years of experience with HIV, who works in San Francisco”.

How much time do we have?

Your: Age, education, years of experience with HIV in the OPD. Days/week, hours/day, pts/hour.

Did my presence change your behaviour?

With each patient, in this consultation:

- Sex, approx. age, RNA, CD4, years with treatment, your time with pt. (treatment).
- Comm.: Can you describe the scenario? What did she say? What did you think / feel / say?
  - I think I heard you saying… “xxx” – comment?
  - Where did you learn to ask like that? Do you facilitate honesty?
  - Barriers (time, shy, not necc.: undetect./resist.) - Facilitators (off-hand talk)
  - Relationship / feelings / years w. pt.. Does that shape adh. comm.?
- *Is this pt. adherent? *What makes you think that? *Why is he that (non-)adherent?
- What did you do to help this patient to be adherent?
- How do you evaluate this work with adherence? What could you have done?
- Has it been different with this patient in the past?
- Is this case typical or no typical? / reminds you of a similar/different case?
- If no med.: Why not on med.’s? Indicated? How do you feel? What did you do / say.

How do you generally assess, explain, enhance and communicate about adherence in:

New-starters (delay?), with suppressed virus, with measurable virus, treatment failure (Stop?),

- Have you viewed this differently in the past?
- (NOT: acknowledge difficult / how many missed / specific time / sit.’s / tricks / importance)
- If a patient is UD, is he then adherent enough?
- Can a non-adherent person be right? (example)

Your vs. routine work of the nurse (or other health personnel).

Is your adherence views different than other’s or colored by some special interests?

Follow up: “Can you remember more details on that case?” Evolving themes followed.

We can finish soon. Comments? Questions? Again: did I change you? You may call me. How was it to participate? Suggestions for the next interview? Thank you!
